# Macrolide-resistant *Bordetella pertussis* strain identified during an ongoing epidemic, Finland, January to October 2024

**DOI:** 10.2807/1560-7917.ES.2024.29.49.2400765

**Published:** 2024-12-05

**Authors:** Milja Miettinen, Alex-Mikael Barkoff, Aino Nyqvist, Carita Savolainen-Kopra, Jenni Antikainen, Jussi Mertsola, Lauri Ivaska, Qiushui He

**Affiliations:** 1 Finnish Reference Laboratory for Diphtheria and Pertussis, Institute of Biomedicine, University of Turku, Turku, Finland; 2 Department of Paediatrics and Adolescent Medicine, Turku University Hospital and University of Turku, Turku, Finland; 3 InFLAMES Research Flagship Centre, University of Turku, Turku, Finland; 4 Department of Public Health, Finnish Institute for Health and Welfare, Helsinki, Finland; 5 HUS Diagnostic Center, Clinical Microbiology, Bacteriology, University of Helsinki and Helsinki University Hospital, Helsinki, Finland

**Keywords:** whooping cough, pertussis, post-COVID-19 resurgence, *Bordetella pertussis*, pertactin expression, macrolide resistance

## Abstract

Since April 2024, a pertussis epidemic has been ongoing in Finland with 2,215 notified cases by end October. Of them, 30.1% (n = 667) were aged 10–14 years. Of the 462 *Bordetella pertussis* isolates characterised, one was macrolide-resistant (minimum inhibitory concentration (MIC) of erythromycin, azithromycin and clarithromycin > 256 μg/mL). The resistant isolate was serotype FIM2, vaccine antigen pertactin-deficient and harboured *ptxP3* allele. The emergence of macrolide-resistant *B. pertussis* in Europe is worrisome and its rapid identification is important.

Whooping cough, or pertussis, is a highly contagious respiratory infection caused by *Bordetella pertussis*. The disease affects people of all ages but can be fatal in infants too young to be vaccinated, in Finland the first dose of pertussis vaccination is given at 3 months of age. Here, we describe an ongoing pertussis outbreak in Finland between April and October 2024 and the detection of macrolide resistance in one isolate.

## Outbreak description

**Figure 1:**
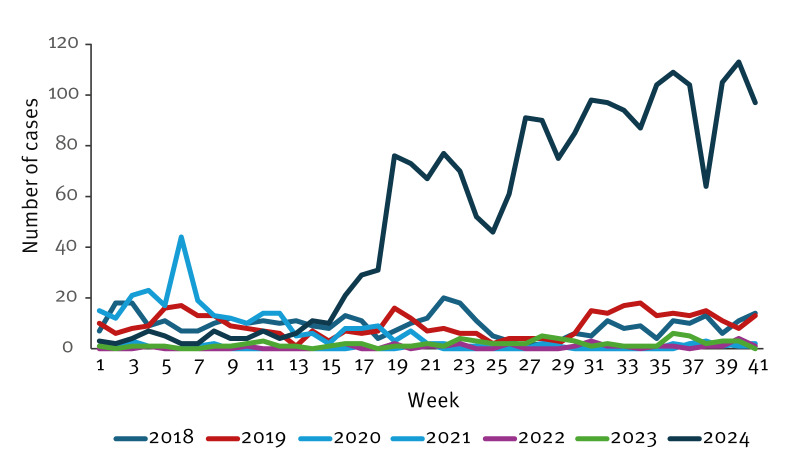
Number of notified pertussis cases per week, Finland, 2018–week 42 2024 (n = 3,741)

**Figure 2:**
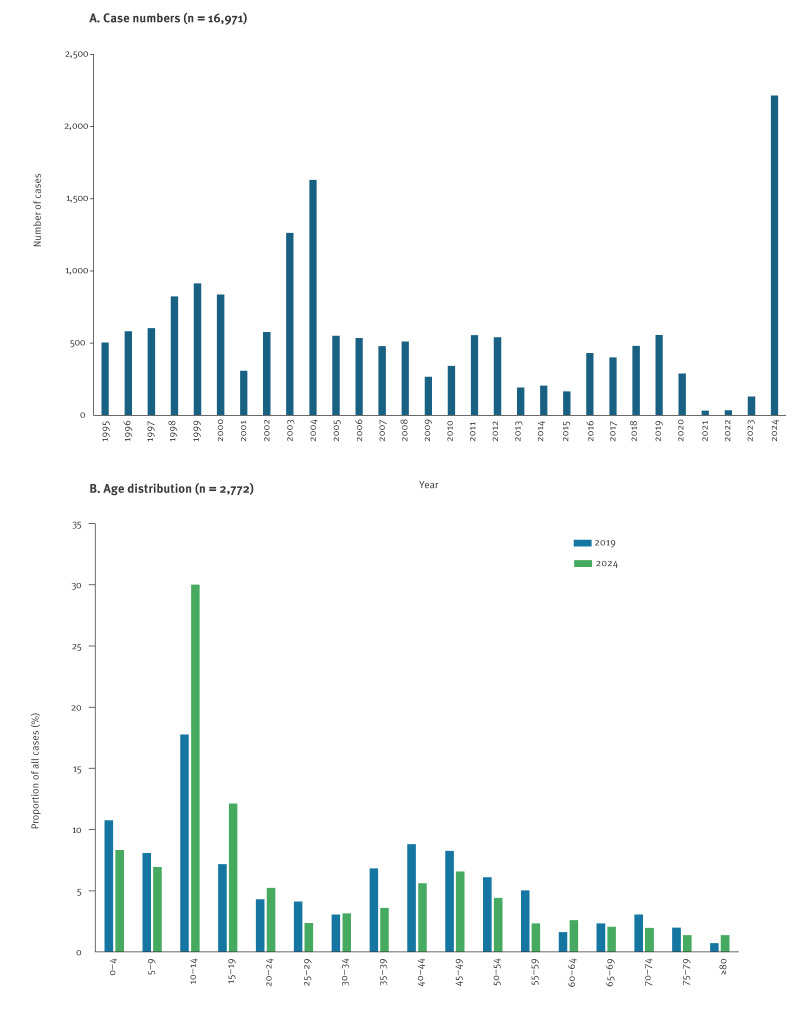
Number of laboratory-confirmed pertussis cases, 1995–October 2024 (n = 16,971) (A) and age distribution of pertussis cases, 2019–October 2024 (n = 2,772) (B), Finland

By the end of October 2024, 2,215 laboratory-confirmed cases were notified, four times more than in the entire year of 2019 (n = 557) (Figure 1, Figure 2A) [1]. In Finland, the European Union (EU) case definition is used [2], and only laboratory-confirmed cases are notified. In 2024, the highest incidence (212.2 cases/100,000 population) was among adolescents aged 10–14 years, with 667 cases (30.1%) notified, followed by 267 cases (88.2 cases/100,000 population) in the age group 15–19 years and 184 cases (75.6 cases/100,000 population) in children aged < 4 years (Figure 2B). In infants aged < 12 months, 75 cases (3.4%) were notified. Of them, 22 were aged < 3 months. From August 2024, the Finnish Institute for Health and Welfare (THL) has recommended a booster vaccination to pregnant people, a preventive measure not previously applied in this country [3].

The outbreak in 2024 started in week 15.

Total number of cases January–October 2024 (n = 2,215).

The map was created with MapChart (https://www.mapchart.net/).

## Diagnosis of pertussis

In Finland, laboratory methods used for diagnosis of pertussis include culture, PCR and serology. Of the 2,215 cases, 886 (40.0%) were diagnosed by PCR, 824 (37.2%) by culture and 505 (22.8%) by serology. Since the National Infectious Diseases Register (NIDR) will only allow one method for each notification, it is possible that most of the culture-positive samples are primarily PCR-positive. This practice has not affected the incidence figures. Compared with years before the COVID-19 pandemic (1995–2019), the corresponding proportions were 11.8% (PCR, n = 1,684), 2.8% (culture, n = 399) and 85.4% (serology, n = 12,185).

**Figure 3:**
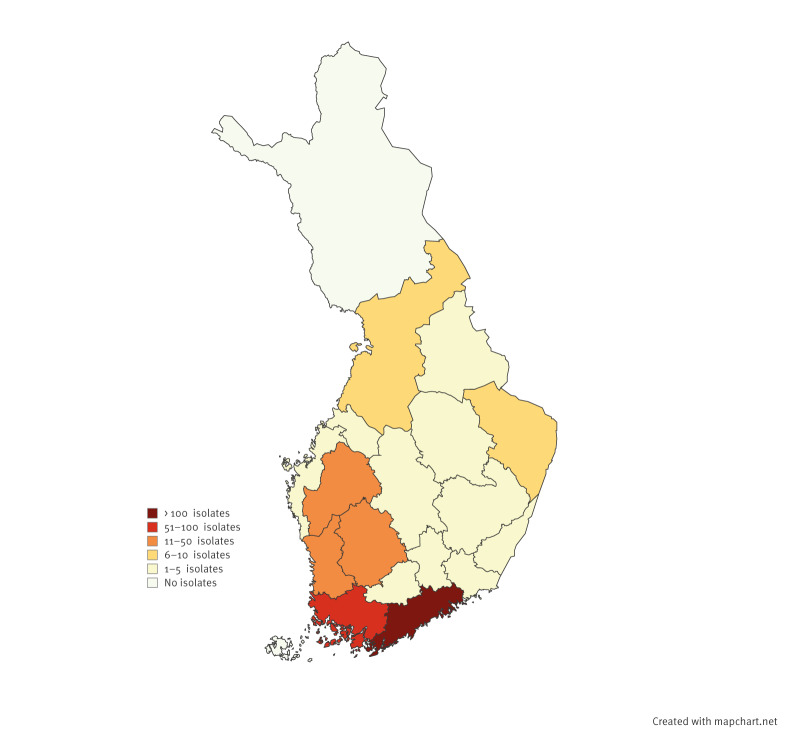
Geographic distribution of *Bordetella pertussis* isolates analysed at the national Reference Laboratory for Diphtheria and Pertussis at the University of Turku, Finland, January–October 2024 (n = 462)

Throughout the country, PCR-positive nasopharyngeal samples and cultured isolates are sent to the Finnish Reference Laboratory for Diphtheria and Pertussis at the University of Turku. By October, we received 462 isolates from different regions of the country (Figure 3). Of them, 195 (42.2%) isolates were collected from males and 265 (57.4%) from females, two samples were unspecified. Most isolates (n = 317; 68.6%) were from the Helsinki metropolitan area, of which 141 (44.5%) were from male and 176 (55.5%) from female patients. The median age of culture-positive patients was 12.9 years (range:  1 month–85 years), with 178 cases (38.5%) in the age group of 10–14 years. There were 24 (5.2%) isolates collected from infants < 12 months and eight (1.7%) from those aged < 3 months.

## Circulating *Bordetella pertussis*

With the introduction of acellular pertussis vaccine (aPv) in 2005, considerable changes have been observed among circulating *B. pertussis*, which include an increase of vaccine antigen pertactin (PRN) deficient isolates and shifts in the distribution of fimbrial (FIM) serotypes. In Finland, almost all *B. pertussis* isolates were of serotype FIM2 before the late 1990s, but FIM3 started to increase from 1999 [4]. Between 2003 and 2004, coinciding with a national epidemic (Figure 2A), FIM3 became dominant, covering > 80% of the isolates [5]. However, from 2011 to 2024, dynamic changes occurred between FIM2 and FIM3 [4]. In 2024, of the 412 isolates tested, 252 (61.2%) were FIM2, 141 (34.2%) FIM3, 16 (3.9%) FIM2,3 and 3 (0.7%) non-typeable.

**Figure 4:**
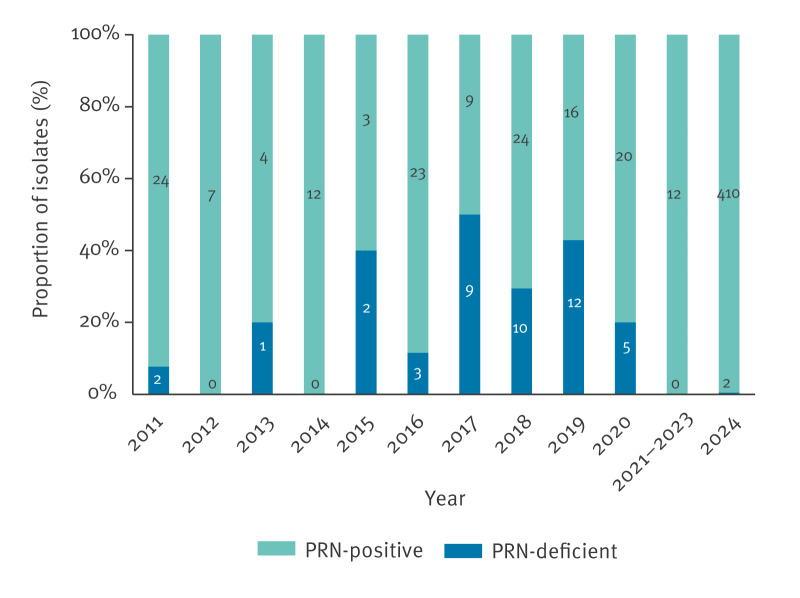
Pertactin-deficient and pertactin-positive *Bordetella pertussis* isolates, Finland, 2011–October 2024 (n = 610)

In Finland, PRN-deficient isolates were first detected in 2011, 6 years after aPV was introduced [6,7]. Since then, the frequency of PRN-deficient isolates has increased (Figure 4). Before COVID-19, i.e. 2011–2019, 39 (24.2%) of 161 characterised isolates were PRN-deficient. Interestingly, during and after COVID-19, the number of PRN-deficient isolates decreased. In 2024, 410 (99.5%) of the 412 tested isolates expressed PRN and only two did not. All 412 isolates expressed pertussis toxin (PT) and filamentous haemagglutinin (FHA). Of the 412 isolates, 238 were genotyped for *ptxP* using a method previously described [8]. Of them, 237 (99.6%) were *ptxP3*, and only one (0.4%) was *ptxP1*.

PRN: pertactin.

The number of PRN-positive and PRN-deficient isolates are given in the bars. Of the 610 isolates, 564 were PRN-positive and 46 PRN-deficient.

## Detection of a macrolide-resistant isolate of *Bordetella pertussis*

So far, the causing mechanism for macrolide resistance in *B. pertussis* is a point mutation A2047G in the 23S rRNA gene within the domain V [9-12]. At the reference laboratory, the methods used to identify the point mutation were block-based allele-specific PCR or High-Resolution Melting Analysis (HRMA) based on real-time PCR [13,14]. For the allele-specific PCR, only one band is observed on agarose gel for resistance, whereas two bands are seen for susceptibility. For HRMA, resistant isolates have a higher melting temperature than the susceptible ones because of the change from A to G in the PCR product. We used Etest (bioMérieux, Marcy-l’Étoile, France) to confirm macrolide resistance as described earlier [15]. A resistant isolate was obtained from a sample of an adolescent male living in the Helsinki metropolitan area. The isolate was resistant to azithromycin (AZT), erythromycin (ERY) and clarithromycin (CHL) based on Etest minimum inhibitory concentrations (MICs) of > 256 μg/mL. It was susceptible to trimethoprim/sulfamethoxazole (SXT), piperacillin-tazobactam (TZP) and ceftazidime (CAZ) with MICs < 0.047 μg/mL, < 0.016 μg/mL and < 0.125 μg/mL, respectively. Cefuroxime (CXM) and doxycycline (DXT) were found to be intermediate, with MIC < 6 μg/mL and < 2 μg/mL. We also examined the other PRN-deficient isolate identified in 2024; it was susceptible to macrolides.

## Discussion

Since April 2024, Finland has been experiencing an ongoing pertussis epidemic with a high number of cases. Compared with age distributions in 2019, i.e. pre-COVID-19, a considerable increase has been observed in age groups of 10–14 and 15–19 years. Unlike many other European countries, we did not see a major increase in infants aged < 3 months (22 cases until the end of October). In Finland, the whole cell pertussis vaccine (wPv) was introduced in 1952. In 2005, wPv was replaced by aPv. Three primary doses are given at 3, 5 and 12 months, followed by three booster doses at ages 4, 14 and 25 years. The coverage of the first three doses has been > 90%. The coverage for the first booster at the age of 4 years has been over 90% (e.g. in 2022: 93.4%). Coverage for the booster at the age of 14 years has been slightly below 90% (e.g. in 2022: 89.4%).

With the non-pharmaceutical interventions implemented during the COVID-19 pandemic, remarkable reductions in pertussis in all age groups were documented globally. However, after interventions related to social distancing were lifted, a resurgence of pertussis has been reported in many countries. In Europe, > 25,000 cases were notified in 2023 and > 32,000 between January and March 2024 [16]. Based on data from European Centre for Disease Prevention and Control (ECDC), the age distribution of pertussis cases also varied between the countries, with 17 European Union/European Economic Area (EU/EEA) countries reporting the highest incidence in infants aged < 1 year. In six other countries, the highest incidence was reported in adolescents aged 10–19 years. It should be kept in mind that among potential factors influencing this are the national differences in testing practices.

We observed a change in the frequency of PRN-deficient isolates pre- and post-COVID-19 pandemic. Before the pandemic, up to 50% of the isolates were PRN-deficient, whereas after COVID-19 almost all produced PRN, supporting the recent finding reported in France [17]. One explanation for the rapid shift from PRN-deficient to PRN-positive isolates might be that since 2019, the acellular vaccine containing only PT and FHA has replaced the aPv containing PT, FHA and PRN for primary vaccinations.

In this study, we identified, to our knowledge, the first macrolide-resistant *B. pertussis* isolate in Finland. The isolate was serotype FIM2 and PRN-deficient and carried *ptxP3* allele, similar to the resistant one in France [17]. Unfortunately, we do not have access to clinical information of the patient, such as travel history and potential treatment with antimicrobials. Previously, macrolide resistance has rarely been reported in Europe, although it is highly prevalent in China [10,18]. However, since 2024, macrolide resistance has been detected in France and Finland [17]. The emergence of macrolide-resistant *B. pertussis* in Europe is worrisome, highlighting the importance of both epidemiological surveillance and diagnostic capabilities to detect resistant isolates rapidly. This is essential especially in infants, in whom early use of effective therapy might reduce the severity and duration of symptoms.

## Conclusion

Our findings emphasise the importance of continuous surveillance to monitor changes in bacterial populations and to study the impact of these changes on the prevention and incidence of pertussis. Clinical microbiology laboratories performing pertussis diagnostics should develop their capabilities to rapidly identify macrolide resistance of *B. pertussis* and thus inform clinical care of the patients.
